# Streptococcus gallolyticus subsp. gallolyticus endocarditis isolate interferes with coagulation and activates the contact system

**DOI:** 10.1080/21505594.2017.1393600

**Published:** 2017-12-26

**Authors:** Julia Isenring, Juliane Köhler, Masanobu Nakata, Marcus Frank, Christoph Jans, Pierre Renault, Camille Danne, Shaynoor Dramsi, Bernd Kreikemeyer, Sonja Oehmcke-Hecht

**Affiliations:** a Institute of Medical Microbiology, Virology and Hygiene, Rostock University Medical Center, Rostock, Germany; b Nutrition and Health, Laboratory of Food Biotechnology, Institute of Food, ETH Zürich, Zürich, Switzerland; c Department of Oral and Molecular Microbiology, Osaka University Graduate School of Dentistry, Suita, Osaka, Japan; d Medical Biology and Electron Microscopy Centre, Rostock University Medical Center, Rostock, Germany; e Micalis Institute, INRA, AgroParisTech, Université Paris-Saclay, Jouy-en-Josas, France; f Unité de Biologie des Bactéries Pathogènes à Gram-positif, Institut Pasteur, Paris, France, Centre National de la Recherche Scientifique (CNRS) ERL3526

**Keywords:** *Streptococcus gallolyticus*, contact system, bradykinin, endocarditis, pili

## Abstract

*Streptococcus gallolyticus subsp. gallolyticus*, formerly classified as *S. bovis* biotype I, is an increasing cause of bacteremia and infective endocarditis in the elderly. The physiopathology of infective endocarditis is poorly understood and involves immune and coagulation systems. In this study, we found that *S. gallolyticus* subsp. *gallolyticus* activates the human contact system, which in turn has two consequences: cleavage of high-molecular-weight kininogen (HK) resulting in release of the potent pro-inflammatory peptide bradykinin, and initiation of the intrinsic pathway of coagulation. *S. gallolyticus* subsp. *gallolyticus* was found to bind and activate factors of the human contact system at its surface, leading to a significant prolongation of the intrinsic coagulation time and to the release of bradykinin. High-affinity binding of factor XII to the bacterial Pil1 collagen binding protein was demonstrated with a K_D_ of 13 nM. Of note, Pil1 expression was exclusively found in *S. gallolyticus* subsp. *gallolyticus*, further supporting an essential contribution of this pilus in virulence.

## Introduction

The opportunistic pathogen *Streptococcus gallolyticus* subsp. *gallolyticus (Sgg)* is asymptomatically found in the gastro-intestinal tract of humans (2.5 – 15%), ruminants and birds. However, *Sgg* is estimated to be the causative agent of endocarditis in 11 – 14% of cases.^1^ The bacteria belong to the S*treptococcus bovis / Streptococcus equinus* complex (SBSEC), which is a highly diverse bacterial group of Gram-positive, non-hemolytic Lancefield group D commensals. The original division of the SBSEC into *S. bovis* and *S. equinus* has further changed over the past years reaching the current splitting into seven main (sub)species, *Streptococcus infantarius* subsp. *infantarius (Sii), Streptococcus lutetiensis, Streptococcus gallolyticus* subsp. *pasteurianus (Sgp), Streptococcus gallolyticus* subsp. *macedonicus (Sgm), Streptococcus gallolyticus* subsp. *gallolyticus (Sgg), Streptococcus alactolyticus* and *S. equinus*.^2–4^ Some species like *Sii* have important impact on African fermented diary food production and Greek cheese production, respectively.^5,6^ In spite of this, several SBSEC members are associated with human infectious diseases such as bacteremia, meningitis, the development of cancer as well as infective endocarditis.^7,8^ Of note, *Sgg* causes 24% of streptococcal endocarditis cases and its incidence is increasing in Europe.^9^ Since the SBSEC is a highly diverse group, common virulence factors are difficult to quote. Surface components, which might contribute to *Sgg* virulence potential, include a polysaccharide capsule and three pili: Pil1, Pil2 and Pil3.^10^ *Pil1* and *pil3* are heterogeneously expressed among the *Sgg* UCN34 population, while *pil2* is expressed at very low level *in vitro*.^11^ Pil1 is constituted of two subunits, the collagen-binding adhesin (Gallo2179) and the major pilin (Gallo2178), which are polymerized by a sortase C enzyme. It was shown that Pil1 is involved in collagen binding, biofilm formation and development of infective endocarditis in rats by colonization of heart valves.^12^ Pil3 was shown to confer adherence to intestinal mucins and thus for colonization of the murine colon.^13^ It was later found that the Pil3 adhesin can also bind to fibrinogen.^14^ Comparative genomics revealed the presence of Pil3 also in *Sii* and *Sgm* whereas Pil1 and Pil2 were not detected in these strains.^15^ This suggests different adhesion abilities of *Sii* and *Sgm* to extracellular matrix proteins in contrast to *Sgg*, which seem to be strain-dependent and potentially positively correlated with blood stream isolation source of SBSEC.^4^

A major player in infective endocarditis is the human coagulation system. The consequences of the interplay between host coagulation factors and microorganisms invading the bloodstream span from septic complications to embolic events.^16^ The coagulation cascade can be activated either by the extrinsic pathway triggered by tissue factor, or the intrinsic pathway, which is induced by binding of the serine protease factor XII (FXII) to a foreign surface followed by its auto-activation. Both pathways result in the activation of thrombin, which leads in turn to the conversion of fibrinogen to fibrin arranged in firm networks.^17^ In infective endocarditis, inflammation, infection and coagulation are deeply intertwined.^18^ Previous studies have shown that prominent pathogens such as *Streptococcus pyogenes or Staphylococcus aureus* trigger activation of the human contact system,^19,20^ also known as the intrinsic pathway of coagulation, further supporting their pathogenic behavior. The contact system comprises the serine proteases factor XI (FXI), FXII, plasma prekallikrein and the co-factor high molecular weight kininogen (HK). Activation of FXII after its binding to a foreign surface initiates the coagulation cascade by activating coagulation factor FXI.^21^ Activated FXII further leads to the conversion of prekallikrein to plasma kallikrein (PK), which in turn positively enhances FXII activation.^22^ PK degrades HK, resulting in the liberation of the pro-inflammatory peptide bradykinin. This peptide, belonging to the family of kinins, induces inflammatory and pain reactions, fever, an increased vascular permeability, vasodilation and the release of proinflammatory mediators.^23^ The present study investigated the ability of *Sgg*, a major causative agent of infective endocarditis in humans, to activate the human coagulation and contact system. We found that *Sgg*, but not *Sii*, is able to activate the contact system at the bacterial surface. Furthermore, we showed that Pil1 associated adhesin is able to bind factor XII with high affinity. Thus, in addition to its ability to bind to host collagen, Pil1 alters host blood coagulation cascade, which might further contribute to the development of infective endocarditis and explain the virulence potential of *Sgg*.

## Results

### Survival of SBSEC strains in human blood and in macrophages

**Figure 1.**
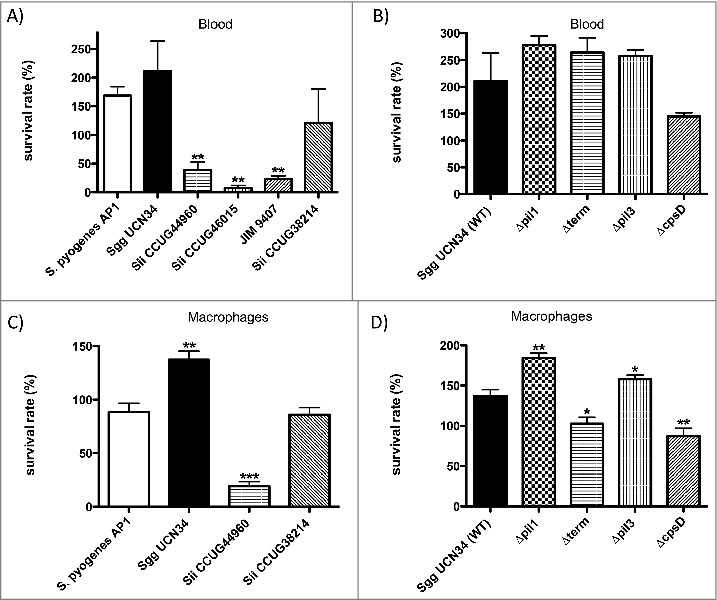
Survival of SBSEC strains in blood and in the presence of macrophages. (A, B) Bacteria (2 × 10^6^ CFU, inoculum) were incubated in citrated blood for 3 hours. Serial dilutions of the samples were plated on BHI agar plates to determine CFU after incubation overnight. (C, D) Bacteria were set to 1 × 10^7^ CFU/ml (inoculum) and preincubated with plasma (1:1). Incubation was followed by the addition of J774 cells (1 × 10^6^ cells/ml). After incubation CFU were determined by plating. Percentage of viable bacteria was calculated in reference to the inoculum. Data represents mean values ± standard deviation, whereas mean values result from three independent biological experiments. Significance values were calculated using the Welch's t-test. * – p < 0.05, ** – p < 0.01, *** – p < 0.001

Survival in human blood is necessary to infect the endocardium. We first compared the survival rate of the *S. gallolyticus* subsp. *gallolyticus* clinical isolate UCN34 (*Sgg* UCN34) with that of four commensal strains of *Streptococcus infantarius* subsp. *infantarius (Sii*) (Fig. 1A). As control, we used the human pathogen *S. pyogenes* AP1, which can survive and multiply in human heparinized blood.^24^ As shown in Fig. 1A, *S. pyogenes* AP1 strain survive and multiply in citrated blood, which is used here as all proteins from the coagulation cascade can be activated. Three *Sii* strains displayed a significant decrease in blood survival but one strain *Sii* CCUG3821 was able to survive and even to multiply (121 ± 60% viable cells). Strikingly, *Sgg* UCN34 showed increased survival and multiplication during incubation time, with 211 ± 52% viable bacteria after 3 h. Survival and growth of this strain in human blood was similar to that of our control strain *S. pyogenes* AP1 (Fig. 1A).

Pili have been identified in *Sgg* UCN34 and both Pil1 and Pil3 have been characterized as important for host colonization. Pil1 binds to collagen type I and was shown to promote heart valve colonization during rat experimental endocarditis.^12^ We next assessed the contribution of Pil1, Pil3 and capsule for the survival of *Sgg* UCN34 in human blood (Fig. 1B). We also included a *ΔcpsD* mutant of *Sgg* strain UCN34, as the gene *cpsD* was previously shown essential for capsule expression in *S. agalactiae*.^25^ These different isogenic mutants (Table 1) were compared to the parental *Sgg* UCN34 for their ability to survive and multiply in human blood. The mutants *Δpil1* and *Δpil3* lack the corresponding *pil* operon, whereas *Δterm* is overexpressing Pil1.^13,26^ The three pilus mutants *Δpil1, Δterm*, and *Δpil3* were almost similar to the wildtype strain. The *ΔcpsD* strain displayed a slightly reduced multiplication in human blood as compared to the wild-type strain, although this difference was not considered statistically significant (Fig. 1B).

**Table 1.** SBSEC strains.

Strain	Source	Origin
*Sii* JIM 9407	Human bacteremia	Spain^47^
*Sii* CCUG38214	Human blood	Sweden
*Sii* CCUG44960	Human blood	Sweden
*Sii* CCUG46015	Human	Sweden
*Sgg* UCN34	Infectious endocarditis and colon cancer	France

To test whether survival of bacteria in whole blood is influenced by phagocytosis, we assessed the capacity of these various strains and mutants to survive phagocytosis and killing by mouse J774 macrophages. The two *Sii* strains, JIM9407 and *Sii* CCUG46015, that showed a very low level of survival in blood, were excluded from this assay. As shown in Fig. 1C, the survival rates of the two tested *Sii* strains correlate with their ability to survive in human citrated blood (Fig. 1C). Interestingly, the survival rate of *Sgg* UCN34 in J774 macrophages is significantly higher when compared to *S. pyogenes* AP1 (Fig. 1C). The non-piliated mutants *Δpil1* and *Δpil3* displayed an increased survival as compared to their wildtype *Sgg* UCN34 (Fig. 1D), whereas the overexpressing Pil1 mutant as well as the *ΔcpsD* mutant were killed more efficiently. Thus, *pil1* and *pil3* expression influence survival rate in the presence of macrophages negatively, whereby the capsule confers the ability to *Sgg* UCN34 to avoid phagocytosis.

### SBSEC strains interfere with coagulation

**Figure 2.**
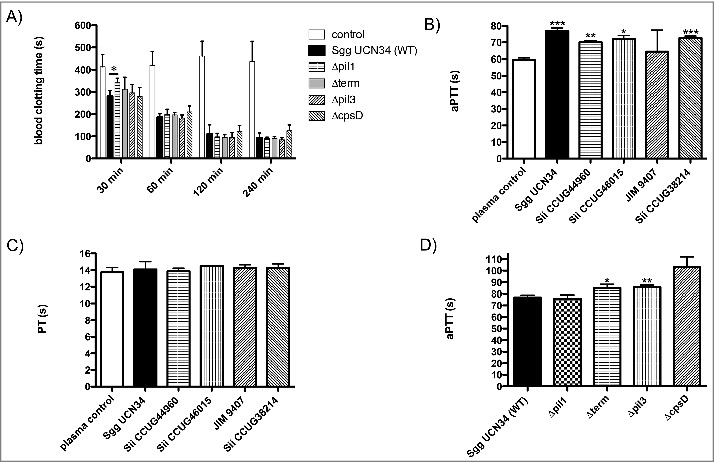
Clotting of blood and plasma after incubation with SBSEC strains. (A) Bacteria (2 × 10^8^ CFU/ml) were added to the same volume of blood. Buffer alone was employed as controls. After incubation for 30, 60, 120 or 240 min at 37°C the recalcification clotting times were measured. (B, C, D) 2 × 10^8^ CFU/ml bacterial overnight cultures were incubated in human plasma for 30 min at 37°C. Plasma incubated with buffer was used as control. Bacteria were removed and the aPTT (B, D) or the PT (C) of the supernatant was determined in a coagulometer. Data represent mean values ± standard deviation, whereas mean values result from three independent biological measurements. Significance values calculated in reference to control using the Welch's t-test. * – p < 0.05, ** – p < 0.01, *** – p < 0.001

Since *Sgg* UCN34 can survive and multiply in human citrated blood, we next examined whether these bacteria could influence the human coagulation cascade. *Sgg* wildtype and mutant strains were incubated with citrated human blood and recalcification clotting times were determined over time period of 240 min. After 30 min of incubation, all *Sgg* strains were able to trigger coagulation, whereby clotting of blood with incubated *Δpil1* mutant strain took significantly longer, compared to the wildtype strain *Sgg* UCN34 (Fig. 2A). This effect was transient, since it was abolished after 60 min of incubation (Fig. 2A). Incubation of bacteria with human plasma instead of human blood did not induce clotting after recalcification (data not shown). The data suggest that Pil1 is involved in induction of blood clotting by *Sgg* UCN34, which is mainly dependent on the cellular components present in the whole blood.

Next, SBSEC strains were incubated with human plasma for 30 min and then removed by centrifugation. Prothrombin time (PT) as well as activated partial thromboplastin-time (aPTT) were determined in the supernatants. Both parameters are commonly used in clinical practice for the global assessment of plasma coagulation. The PT evaluates the extrinsic coagulation pathway, whereby FXII activation is the mechanistic basis for the aPTT.

Significantly prolonged aPTT (Fig. 2B) but not PT (Fig. 2C) values were observed for all bacterial strains (except *Sii* JIM 9407) as compared to plasma samples incubated with buffer alone (Fig. 2B). This result strongly suggests that *Sgg* and *Sii* can bind factors involved in the intrinsic pathway of coagulation on their surface. Mutants lacking Pil3 or overexpressing Pil1 increased the aPTT significantly (8.96 and 8.25 seconds, respectively, see Fig. 2D). This indicates that Pil1 and Pil3 of the *Sgg* UCN34 wildtype are involved in the interaction with the intrinsic pathway of coagulation.

### Sgg UCN34 binds and activates FXII/PK at the bacterial surface

**Figure 3.**
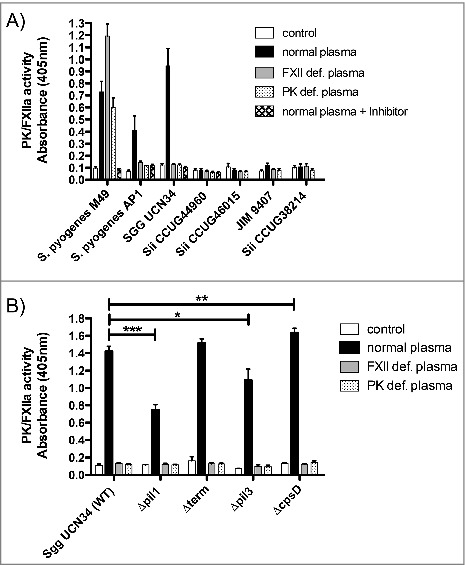
Activation of FXII/PK on the bacterial surface of 5 SBSEC strains (A) and Sgg UCN34 and its mutant strains (B). Bacteria were incubated in HEPES buffer (neg. control), normal human plasma, PK- and FXII-deficient plasma or normal human plasma preincubated with the inhibitor H-D-Pro-Phe-Arg-CMK. After a washing step, bacteria were incubated with the chromogenic substrate S-2303 and absorbance at 405 nm was determined. Data represents mean values ± standard deviation, whereas mean values result from three independent biological measurements. Significance values were calculated in reference to the control using the Welch's t-test. * – p < 0.05, ** – p < 0.01, *** – p < 0.001

We then asked if these bacteria could activate the human contact system. To answer this question, bacteria were incubated with human plasma, washed, and activation of contact factors at the bacterial surface was assessed using a specific chromogenic substrate, detecting activity of FXII and PK. *S. pyogenes* M49 and AP1 strains served as positive controls. Bacteria incubated with buffer only served as negative control, to exclude activation of the substrate by bacterial components. PK- and FXII- deficient plasma, respectively, were used as negative controls, since in these plasmas the corresponding factor is missing, no activity should be observed. *S. pyogenes* M49 is an exception in this case, as its abundant formation of streptokinase leads to plasmin and subsequent FXII and/or PK activation.^19^ Therefore, the inhibitor H-D-Pro-Phe-Arg-chloromethylketone (CMK) peptide was additionally used, which inhibits both PK and FXII.^27^ *Sgg* UCN34 showed a high FXII/PK activity at the bacterial surface as compared to the other *Sii* strains, that was even higher than in the control *S. pyogenes* AP1 (Fig. 3A). Comparing *Sgg* UCN34 to *S. pyogenes* M49, no FXII/PK activity was detectable when the bacteria were incubated in the corresponding deficient plasmas (Fig. 3A).

Next, we tested the ability of *Sgg* mutants to induce human contact system activation (Fig. 3B). The pilus mutants *Δpil1* and *Δpil3* showed significantly decreased activation of FXII/PK compared to the wildtype. On the other hand, higher activity of FXII/PK was observed with the *ΔcpsD* mutant, which is in agreement with the highest aPTT prolongation time (Fig. 2C).

To determine whether *Sgg* UCN34 secretes components involved in human contact activation, bacterial supernatants were added to plasma and FXII/PK activity was measured, but no activation could be detected (data not shown).

### Plasma proteins aggregate at the bacterial surface

**Figure 4.**
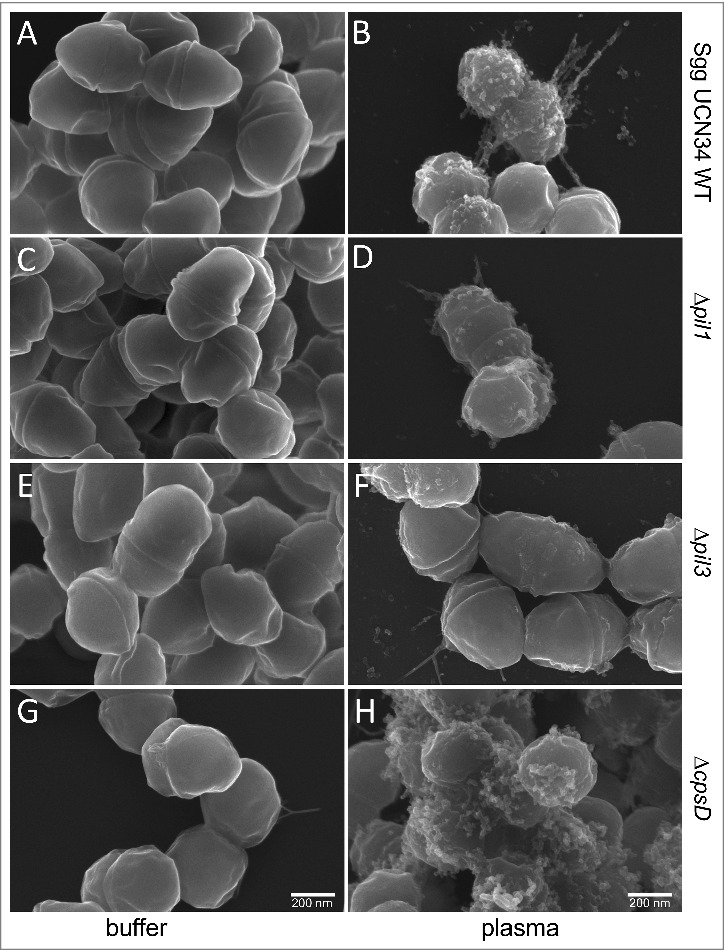
Scanning electron microscopy of Sgg UCN34 and its mutants incubated with plasma. Representative scanning electron micrographs of bacteria incubated in plasma or PBS. (A) Sgg UCN34 wildtype in PBS, (B) Sgg UCN34 wildtype in plasma, (C) *Δpil1* in PBS, (D) *Δpil1* in plasma, (E) *Δpil3* in PBS, (F) *Δpil3* in plasma, (G) *ΔcpsD* mutant in PBS (H) *ΔcpsD* mutant in plasma. Scale bars represent 200 nm.

It is known that *S. pyogenes*, when exposed to plasma, forms a dense plasma protein layer around its surface.^28^ The interaction of *Sgg* UCN34 with plasma has not been studied yet, which prompted us to employ scanning electron microscopy (SEM) to analyze the morphology of *Sgg* UCN34 wildtype and mutants after incubation with PBS or human plasma. As shown in Fig. 4, bacteria incubated in PBS appear smooth (Fig. 4A, 4C, 4E, 4G), whereas plasma-incubated bacteria exhibit aggregates distributed unevenly at the surface. Thus, in contrast to *S. pyogenes, Sgg* is not coated by an additional dense layer of plasma components. Instead some bacteria displayed pilus-like structures, which seem to be covered by plasma proteins (Fig. 4B, 4D). Such aggregates were found in the wildtype (Fig. 4B) and the *ΔcpsD* mutant (Fig. 4H), and to a lower extent at the surface of the *Δpil1*-mutant (Fig. 4D) and *Δpil3* mutant (Fig. 4F) strains. Thus, assembly of plasma components at the bacterial surface seems to be supported by pili in *Sgg* UCN34, and the absence of the capsule enhance this effect probably by unmasking the pili at the bacterial surface.

### Detection of HK and its degradation products bound to the bacterial surface

**Figure 5.**
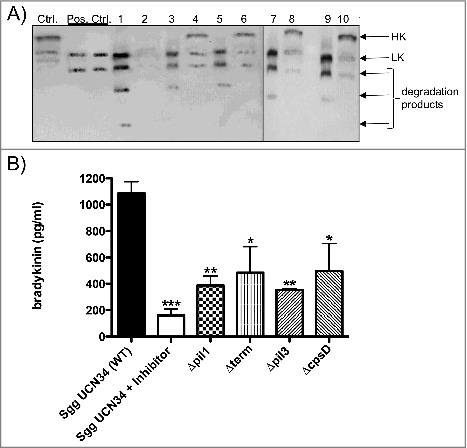
Cleavage of HK at the surface of *Sgg UCN34* and release of bradykinin. (A) Bacteria (10^8^ CFU/ml) were incubated with human plasma for 15 min. After washing, bacteria-bound proteins were eluted with a glycine buffer (eluate). Eluate and supernatants were then separated on SDS-PAGE, transferred to Immobilon filters, and immunostained with a polyclonal antibody against HK. Ctrl: non-activated plasma; pos. Ctrl.: plasma activated with DAPPTIN; Lane 1: eluate of Sgg UCN34; lane 2: supernatant of Sgg UCN34; lane 3: eluate from *Δpil1* mutant; lane 4: supernatant from *Δpil1* mutant; lane 5: eluate from *Δterm*; lane 6: supernatant from from *Δterm*; lane 7: eluate from *Δpil3* mutant; lane 8: supernatant from *Δpil3* mutant; lane 9: eluate from *ΔcpsD* mutant; lane 10: supernatant from *ΔcpsD* mutant. B) Bacteria were incubated in plasma, washed, and further incubated in HEPES for 15 min. After centrifugation, bradykinin was determined in the supernatant. Data represents mean values ± standard deviation, whereas mean values result from three independent biological measurements. Significance values were calculated in reference to Sgg UCN34 using the Welch's t- test. * – p < 0.05, ** – p < 0.01, *** – p < 0.001

We next analyzed the binding and degradation of HK at the surface of *Sgg* UCN34 and its mutant strains by Western Blot and immunoprinting (Fig. 5A). Plasma-supernatants recovered after bacterial incubation were also analyzed. Plasma alone or plasma treated with DAPPTIN (a contact activator) were used as negative and positive control, respectively. Immunoblotting was performed using antibodies directed against HK and low-molecular weight kininogen (LK). Notably, LK is a shorter splice variant of HK^29^ and the polyclonal antiserum against HK also reacts against LK. Fig. 5A depicts intact HK at 120 kDa and LK at 66 kDa. As shown in Fig. 5A, the HK protein has been processed after DAPPTIN treatment (pos. ctrl.) and a similar pattern was observed with all the eluate samples, which contain plasma proteins absorbed from the surface of *Sgg* UCN34 and its mutants (Fig. 5A, lanes 1, 3, 5, 7, 9) indicating that they are all able to bind and degrade HK on their surface. Of note, the 120 kDa HK signal is completely absent in the plasma-supernatant of *Sgg* UCN34 (Fig. 5A, lane 2) whereas full-length HK is still detectable in the plasma-supernatants of the various *Sgg* mutants (Fig. 5A, lanes 4, 6, 8, 10). These results indicate that only *Sgg* UCN34 wildtype has the capacity to absorb HK completely from human plasma.

Moreover, in the eluate of the *Sgg* UCN34 wildtype (Fig. 5A, lane 1) an additional 20 kDa degradation product was detected, which supports a further processing of HK that did not occur in any other mutant strain.

We next investigated the release of bradykinin from the bacterial surface of *Sgg* UCN34 wildtype and mutant strains by ELISA.^20^ The inhibitor H-D-Pro-Phe-Arg-CMK was added to the plasma of the wildtype sample to test whether bradykinin formation is dependent on PK and FXII. *Sgg* UCN34 wildtype leads to the release of about 1,1 ng of bradykinin per ml (Fig. 5B). For all mutants, significantly lower concentrations of bradykinin were detected, whereas the two pilus mutants showed the most significant decrease (Fig. 5B). Addition of the PK/FXII inhibitor H-D-Pro-Phe-Arg-CMK to the plasma of the wildtype strain sample revealed significantly diminished bradykinin levels, showing that FXII and PK play a major role in bradykinin release from *Sgg* surface (Fig. 5B). Altogether, we showed that binding and degradation of HK occurs from the surface of the *Sgg* UCN34 wildtype well as the mutant strains, but the wildtype strain absorbed HK completely from plasma and released highest amounts of bradykinin.

### Pil1 of Sgg UCN34 binds FXII

**Figure 6.**
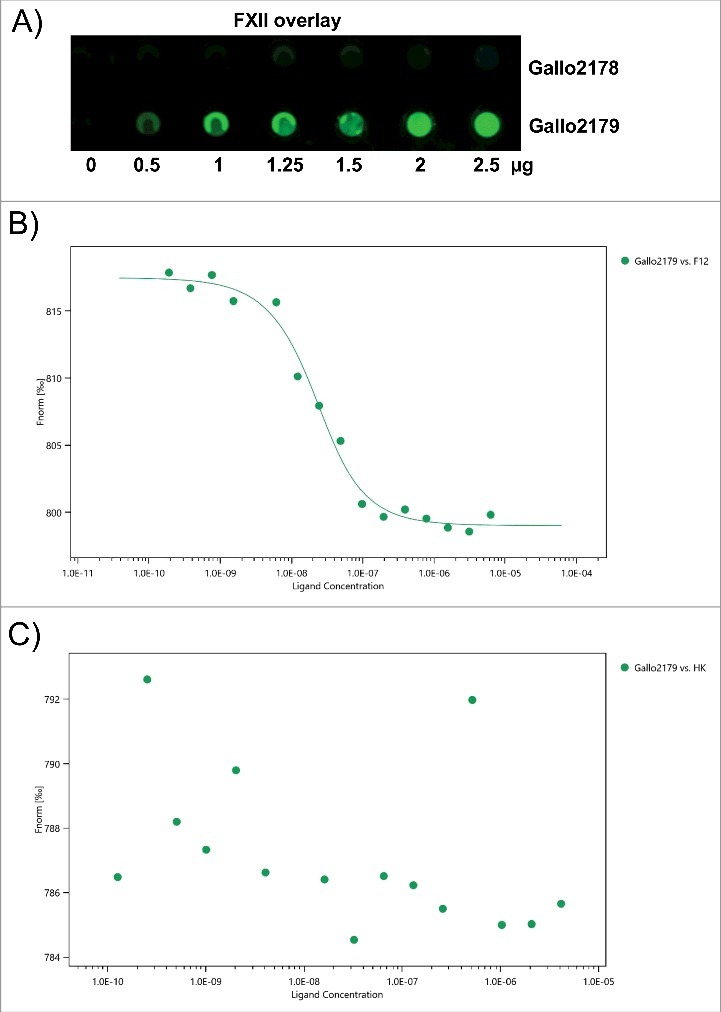
Binding of FXII to recombinant pilus protein. (A) Dot blot overlay was performed after immobilization of recombinant *Sgg* UCN34 pili proteins Gallo 2178 or 2179 in amounts of 2.5 µg, 2 µg, 1.5 µg, 1.25 µg, 1 µg and 0.5 µg onto a nitrocellulose membrane. Human FXII was used for protein overlay. Binding was detected with polyclonal FXII-specific antibodies and Irdye labeled secondary antibody followed by fluorescence detection. (B, C) A fluorescent label (NT-647) was covalently attached to Gallo2179 protein (NHS coupling). The concentration of NT-647 labeled Gallo2179 was kept constant, while the concentration of the non-labeled molecule FXII or HK varied between 6.25 μM – 0.19 nM (B) or 4.15 μM – 0.12 nM (C). Concentrations on the x-axis are plotted in nM. A Kd of 12.89 nM was determined for the interaction of FXII with Gallo2179 (B).

As deletion of Pil1 had the strongest influence on activation of FXII/PK (see Fig. 3B), we investigated whether Pil1 proteins could bind HK, PK or FXII. Recombinant pili proteins were immobilized in different amounts (0.5 µg, 1 µg, 1.25 µg, 1.5 µg, 2 µg and 2.5 µg) on a membrane. Human PK, HK or FXII were used for protein overlay and binding was detected with specific antibodies. Results of the overlay demonstrated that the Pil1 adhesin Gallo2179 binds to FXII (Fig. 6A), whereas no binding to PK or HK could be detected (data not shown). In contrast, the Pil1 major pilin Gallo2178 did not bind FXII (Fig. 6A) nor PK or HK (data not shown).

Binding of Gallo2179 to FXII and HK were further investigated by the microscale thermophoresis (MST) method.^30^ Based on a different thermophoresis of a protein after binding to an unlabeled interaction partner, it is possible to determine the dissociation constant (K_D_). Gallo2179 binding to FXII was confirmed in a dose-dependent manner using concentrations of the non-labeled molecule FXII between 6.25 μM and 0.19 nM (Fig. 6B). A K_D_ of about 13 nM was determined for this interaction. HK did not bind to Gallo2179 by this method (Fig. 6C), which supports results from dot blot analysis. MST does not provide information on reaction kinetics, thus we additionally employed surface plasmon resonance to determine association and dissociation rate constants (suppl. Table 1). Collagen I was used as a positive control, as Gallo2179 is responsible for *Sgg* adhesion to collagen I.^12^ For the interaction between Gallo2179 and FXII the K_D_ in a double-digit nanomolar range (14 nM) was verified with this method (suppl. Table 1).

### Pil1 is not present in Sii strains

**Figure 7.**
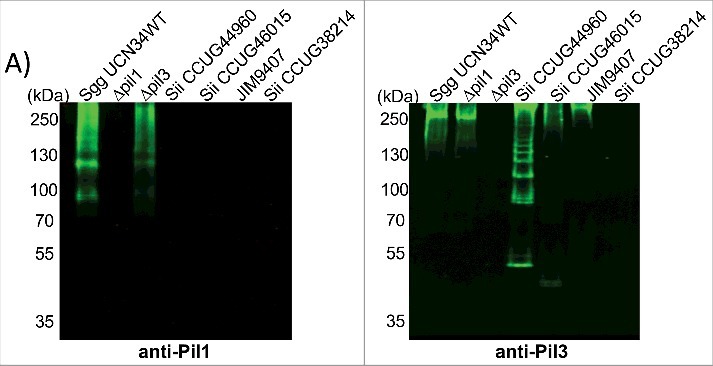
Pilus polymerization in SBSEC strains. Western blot analysis of cell wall protein extracts isolated from *Sgg* UCN34, *Δpil1* mutant, *Δpil3* mutant and *Sii* strains separated with 4%–12% Criterion XT sodium dodecyl sulfate–polyacrylamide gel electrophoresis and detected by means of immunoblotting using specific anti-Pil1 (left) or anti-Pil3 (right) polyclonal antibodies (pAbs). Equivalent amount of total proteins was loaded in each well.

Combined with the results described above, it is possible that *Sii* strains bind factors involved in the intrinsic coagulation pathway on their surface, but not FXII or PK. Interestingly, the *pil1* locus of *Sgg* UCN34 is present in 90% of *S. gallolyticus* infective endocarditis clinical isolates, but absent in the closely related species *Sii* and *Sgm* which only harbor a *pil3* locus.^12^ As it is not known whether *Sii* strains, used in this study as comparison SBSEC strains, produce Pil1 or Pil3 type pili, we assessed Pil1 and Pil3 biogenesis by Western blotting of cell wall protein extracts from *Sgg* and *Sii* strains using specific antibodies directed against the Pil1 major pilin Gallo2178 (anti-Pil1) or the Pil3 major pilin Gallo2039 (anti-Pil3).^12^ The antiserum against Pil1 recognized high-molecular-weight species with the typical laddering profile in cell wall protein extracts from *Sgg* UCN34 and *Δpil3* but not *Δpil1* mutant strains (Fig. 7A). None of the 4 *Sii* strains that we tested did react with anti-Pil1 (Fig. 7A). In contrast, 3 of 4 *Sii* strains did react with anti-Pil3, suggesting that some *Sii* produce Pil3 pili but not Pil1 pili, as previously indicated.^15^ However, the expressed Pil3 pili are apparently not able to bind and activate the contact system. Together, our data imply an important role for Pil1 in binding and activation of FXII by *Sgg* UCN34 strain.

## Discussion

Infective endocarditis causative pathogens affect host blood coagulation by binding factors of the hemostasis system, such as fibronectin, collagen and fibrinogen, by activation of platelets or secretion of proteases that activate proenzymes of blood coagulation and fibrinolysis system.^16^ Furthermore it has been shown that a patient with *Sgg* induced infective endocarditis suffer from disseminated intravascular coagulation (DIC).^31^ In the present study, we report that *Sgg*, a leading cause of streptococcal endocarditis in the elderly, triggers coagulation of blood and activates the human contact system at the bacterial surface. Contact activation is a common trait among other human pathogens^32^ and assembly of contact factors on bacterial surfaces is mandatory for activation.^33^ Three of four investigated *Sii* strains significantly prolonged the aPTT, indicating that these strains bind host factors involved in the intrinsic coagulation pathway, depleting them in plasma and causing therefore a prolonged clotting time. As Pilus 3 is expressed in 3 of our *Sii* strains (this study) and was described to bind fibrinogen,^14^ fibrinogen binding could be a reason for prolonged aPTT. However, the prothrombin time was not influenced by *Sii* and *Sgg* strains, indicating that a possible fibrinogen binding by these strains does not influence clotting. Furthermore, no activation of FXII/PK could be detected on *Sii* strains, implicating no binding. In contrast, the SBSEC member *Sgg* UCN34 can bind and activate FXII/PK, even more potently than the human pathogen *S. pyogenes.*

Our results support the involvement of Pil1 and Pil3 in contact activation by *Sgg* UCN34, with Pil1 playing a major role. To date, assembling of contact factors on fibrous bacterial surface proteins such as curli or fimbriae has been described only for Gram-negative *Escherichia coli* and *Salmonella typhimurium.*^33^ The Gram-positive *S. pyogenes* binds HK via its surface M protein, which appears to form hair-like structures at the bacterial surface,^34^ but is not part of a pilus structure.^35^ The fibrinogen-binding M-like protein FOG – a surface protein in Group G streptococci – also binds HK, FXII and FXI.^36^ In contrast to M protein and FOG, here we show that the Pil1 adhesin of *Sgg* UCN34 only binds contact factor XII with high affinity, but not PK or HK, implicating that other surface proteins must recruit PK and HK to the bacterial surface.

Binding and degradation of HK at the bacterial surface was shown by Western blot analysis and bradykinin ELISA. Interestingly, the overexpressing Pil1 mutant as well as the *ΔcpsD* mutant, which showed both higher activation of FXII/PK in comparison to the wildtype strain, had significantly less bradykinin concentration in the supernatant than *Sgg* UCN34. Lack of the polysaccharide capsule may lead to more direct exposure of surface structures, which are then able to interact more intensively with plasma proteins. This adhesive surface may lead to a strong binding, aggregation and activation of FXII on the bacterial surface. This idea is supported by morphological analysis with SEM in our study, showing large aggregates on the bacterial surface of wildtype and *ΔcpsD* mutant bacteria. Cleavage products of HK, such as bradykinin or antimicrobial peptides,^37^ may stick to the bacterial surface and will therefore not be detected in high amounts in the supernatant.

Since the contact system constitutes a link between inflammation and coagulation, disturbance of this equilibrium could trigger infectious diseases. Thus, our findings could represent a novel pathway for causing or interfering with infective endocarditis. We propose the following scenario to explain how *Sgg* triggers infective endocarditis: (I) survival and multiplication of *Sgg* in human blood after entering the bloodstream (II) activation of cellular components of the coagulation cascade and induction of a procoagulant state (III) adherence to collagen on heart valves mediated by Pil1^12^ (IV) binding and activation of contact factors at the bacterial surface (V) bradykinin release and binding of bradykinin to its receptor B2R may trigger infective endocarditis. Of note, receptor B2R is found in the endocardium of atria, atrioventricular valves, and ventricles.^38^ Bradykinin is one of the most potent inflammatory mediators we have in the human body,^39^ it helps in recruitment of neutrophils and monocytes and boost neutrophil activation.^40^ It remains to be investigated whether these events take place *in vivo*, but if so, interfering with contact activation^41^ would be an attractive target for treatment. Altogether, both – coagulation and contact activation by *Sgg* trigger inflammation and may explain the association of these bacteria with infective endocarditis.

## Material and methods

### Bacterial strains and culture conditions

Bacterial strains are listed in Table 1. *Sii* strains were isolated from human origins in Sweden and Spain. Wildtype *Sgg* UCN34 was isolated at the Hospital in Caen (Calvados, France). Mutants of this strain were previously described^12,26^ and are listed in Table 2. We generated a CpsD (gallo_0947) mutant, as the gene *cpsD* was previously shown essential for capsule expression in *S. agalactiae*.^25^ *S. pyogenes* strain AP1 is a *covS* truncated clinical isolate of the M1 serotype strain 40/58 from the WHO Collaborating Centre for Reference and Research on Streptococci, Prague, Czech Republic, and serotype M49 strain 591 was obtained from R. Lütticken (Aachen, Germany). Bacteria were grown on blood agar plates at 37°C, aerobically, overnight and subsequently stored at 4°C. For further use, overnight cultures were cultivated in BHI broth at 37°C under a 5% CO_2_^–^20% O_2_ atmosphere, centrifuged (2500 *g*, 5 min), washed twice in Phosphate Buffered Saline and set to the desired CFU/ml.

**Table 2.** Mutants of the *Sgg* UCN34 wild type strain.

Mutant	Description	Reference
*Δpil1*	*Δpil*1 mutant	^26^
*Δterm*	*pil1* overexpressing mutant	^11^
*Δpil3*	*Δpil3* mutant	^13^
*ΔcpsD*	*ΔcpsD* mutant	this work

### Material

VisuCon-F Frozen Normal Plasma (Haemochrom Diagnostica, Germany) contains pooled citrated human plasma from at least 20 healthy donors. Prekallikrein and FXII deficient human plasma from human donors with a congenital deficiency were from George King Biomedical, Inc. (Overland Park, Kansas).

### Survival in whole blood

Survival in human citrated blood was performed as previously described.^42^ Briefly, bacterial strains were grown to mid-log exponential growth phase (OD_600nm_ = 0.3), harvested by centrifugation and set to 1*10^8^ CFU/ml in PBS. CFU of this suspension were determined by plating serial dilutions (inoculum). Further, 20 µl thereof were inoculated with 480 µl of citrated blood to a final bacterial count of 5*10^3^ CFU/ml. After incubation at 37°C with rotation for 3 h, the CFU were determined by plating and then related to the inoculum. Blood survival was performed with blood samples from three volunteers.

### Phagocytosis assay

The phagocytosis assay was performed as described previously^43^ with minor modifications. Shortly, bacteria were grown to mid-exponential growth phase (OD_600nm_ = 0.3), washed twice in PBS and set to 1*10^7^ CFU/ml. Bacteria were then preincubated with plasma (1:1) at 37°C, 30 min with gentle agitation. Incubation was followed by the addition of J774 cells (1*10^6^ cells/ml) and DMEM as control, respectively. After further incubation for another 30 min at 37°C, phagocytosis was stopped by the addition of ice-cold PBS and the suspensions were centrifuged (5 min, 425 *g*, 4°C). For the determination of intracellular bacteria, the pellet was washed twice with PBS (5 min, 425 *g*, 4°C), resuspended in 1 ml ddH_2_O for cell lysis and subsequently plated on BHI agar. Bacteria from the control tube were harvested, washed and plated as well. CFU were determined for intracellular and the control group bacteria, whereas the control served as reference value for the calculation of the survival rate.

### Clotting assays

Mid-log phase bacteria were washed twice with HEPES buffer (115 mM NaCl, 20 mM HEPES) and resuspended in HEPES buffer. 480 µl bacteria (2 × 10^8^ CFU/ml) were added to the same volume of blood. Buffer alone were employed as controls. After incubation for 0.5, 1, 2 or 4 h at 37°C, 50 µl 25 mM CaCl_2_ were added to 50 µl of the samples, and the recalcification clotting times were measured in a semi-automatic ball coagulometer (MERLINmedical Coagulometer, ABW Medizin und Technik GmbH, Germany).

For measurement of the activated partial thromboplastin time (aPTT) bacterial overnight cultures were set to 2*10^8^ CFU/ml in HEPES buffer. 200 µl of the suspension was added to 200 µl human plasma and incubated for 30 min at 37°C. Plasma incubated with buffer was used as control. Bacteria were removed by centrifugation (3400 *g*, 15 min) and 50 μl of the supernatant was incubated in the coagulometer at 37°C for 60 s, followed by the addition of the same amount of DAPTTIN (Haemochrom Diagnostica, Germany) and further incubation at 37°C for 60 s. Clot formation was initiated by addition of 50 µl CaCl_2_ (30 mM) and clotting time was measured. The PT was measured by incubation of 50 µl supernatant at 37°C for one minute, followed by the addition of the same amount of PT reagent (Haemochrom Diagnostica, Germany) and subsequent clotting time measurement. Experiments were repeated three times and three replicates per repetition were measured.

### Chromogenic substrate assays

For evaluation of FXII/PK activation at the bacterial surface, overnight cultures were set to 2*10^8^ CFU/ml in HEPES and 100 µl of the suspension was added to equal amounts of normal plasma, deficient plasmas as indicated or HEPES as control. Additionally, the inhibitor H-D- Pro-Phe-Arg-chloromethylketone trifluoroacetate (H-D-Pro-Phe-Arg-CMK, Bachem AG, Switzerland), which inhibits PK and FXII, was preincubated with plasma. After incubation for 30 min at 37°C, bacteria were washed three times, resuspended in 300 µl HEPES and supplemented with 100 µl of the chromogenic substrate S-2302 (4 mM, Haemochrom Diagnostica, Germany). After incubation for 60 min at 37°C, cells were removed by centrifugation and absorbance was measured at 405 nm.

Measurement of contact system activation by bacterial supernatants was performed by incubation of 100 µl supernatant from overnight cultures together with 100 µl of plasma and chromogenic substrate S-2302 (1 mM). Plasma mixed with medium served as control. After incubation at 37°C for 1 h the absorbance was determined at 405 nm.

### Bradykinin-ELISA

For sampling, overnight cultures were set to 2*10^8^ CFU/ml in HEPES buffer and 250 µl thereof were incubated at 37°C for 15 min with equal amounts of human normal plasma. Additionally, the inhibitor H-D-Pro-Phe-Arg-CMK was incubated with plasma and the WT strain. Incubation was followed by centrifugation (6800 *g*, 5 min), washing twice in HEPES and resuspension of the pellet in 150 µl HEPES. After further incubation for 15 min and 30 min, respectively, samples were centrifuged (12000 *g*, 5 min) and bradykinin contents in the supernatant were determined as described earlier.^44^ Samples were collected from three independent experiments.

### Electrophoresis and Western blot analysis

For sampling, overnight cultures were set to 2*10^8^ CFU/ml and 250 µl were mixed with equal amount of human normal plasma and incubated at 37°C for 15 min with shaking (600 rpm). Incubation of plasma with PBS, bacteria with PBS and plasma with DAPTTIN served as controls. After centrifugation (6800 *g*, 5 min), 2 µl of supernatants was supplemented with 98 µl SDS sample buffer. Pellets were washed 3 times (6800 *g*, 5 min) in PBS, resuspended in 100 µl glycine (0.1 M) and incubated at room temperature for another 10 min. The pH value of supernatants from subsequent centrifugation (12000 *g*, 5 min) was neutralized by the addition of 20 µl Tris-HCl (1 M, pH = 8.4) and 100 µl of the suspensions was mixed with 20µl SDS sample buffer (5x). Sampling was performed on three different days. SDS-PAGE was performed as described earlier.^45^ Following SDS-PAGE, separated proteins were transferred onto nitrocellulose membranes. Western blot analyses were performed with sheep antibodies against HK (Affinity Biologicals) and its degradation products as described previously.^19^

### Dot-blot overlay

Recombinant pili proteins were immobilized on a nitrocellulose membrane using 1.25, 2.5, 5 and 10.0 μg of protein. Unspecific binding sites were blocked by incubation with Odyssee Blocking buffer (LI-COR Biotechnology – GmbH, Germany). After 3 washing steps with PBST, the membrane was incubated with 50 μg FXII, HK or PK (in 4 ml PBST, Haemochrom Diagnostica, Germany) overnight at 4°C. The following primary antibodies were employed: polyclonal sheep antibodies against HK (Affinity Biologicals), polyclonal rabbit antibodies against FXII or PK (Santa Cruz Biotechnology). The blots were developed using Irdye labeled secondary antibodies and LI-COR reagents for an Odyssey® Infrared Imaging System (LI-COR Biotechnology – GmbH, Germany).

### MST analysis

For MST measurements, 20 µM recombinant Gallo2179 was labeled with fluorescent red dye NT-647 (labeling kit by NanoTemperTechnologies). Labeled Gallo2179 and factor XII or HK (Haemochrom Diagnostica, Germany) were applied to the Monolith NT.115 (NanoTemper Technologies GmbH, München, Germany). For K_D_ value determination, a serial dilution of 16 dilutions of the non-labeled ligand (FXII or HK) was prepared. The concentration of the fluorescently labeled Gallo2179 was kept constant at estimated 50–300 nM and the concentration of the ligand was varied. In the dilution series, the highest concentration was chosen to be at least 20-fold higher than the expected K_D_. 10 μl of the serial dilution of the non-labeled molecule were mixed with 10 μl of the diluted fluorescently labeled Gallo2179. Mixed samples were loaded into glass capillaries and the MST analysis was performed using the Monolith NT.115. The K_D_ value was determined from three independent measurements using MO Affinity Analysis v2.1.3 software (NanoTemper Technologies GmbH). The software calculates the extent of binding by plotting the ratio between the fluorescence when the laser is on and the fluorescence before the laser is turned on. As each curve represents a different concentration of binding partner, these ratios are plotted as a function of binding partner concentration to give a binding curve.

### Surface plasmon resonance

The interactions between FXII or Collagen I (as analytes) and Gallo2179 (as a ligand) were analyzed with a Biacore 3000 system (Biosensor, La Jolla, Calif., USA) as described before.^46^ Briefly, CM3 sensor chips at 25°C in PBS as running buffer were used. Gallo2179 was immobilized (1900 RUs) on the flow-cell surface of the chip using standard amine-coupling chemistry and the software tool ‘Application Wizard-Surface Preparation’ (Biacore 3000 instrument handbook). Each analyte-ligand complex was allowed to associate and dissociate for 3 and 5 min, respectively, with background subtraction using a flow cell that was subjected to the coupling reaction but without protein, as a reference surface. For concentration series, FXII and Collagen I were tested at concentrations between 12.5 – 200 nM. The ligand surface was regenerated with a 15-second injection of 0.5% SDS at the end of each binding cycle. The data from the Biacore sensorgrams were fitted globally, using the 1-step biomolecular association reaction model (1:1 Langmuir binding).

### Scanning electronic microscopy

For sampling, overnight cultures were set to 2*10^8^ CFU/ml in HEPES buffer and incubated in equal amounts of human normal plasma at 37°C for 15 min. Incubation was followed by washing twice in HEPES and resuspension of the pellet in 2.5% (v/v) glutaraldehyde in sodium phosphate buffer for at least 24 h, then washed with sodium phosphate buffer. Sample aliquots were attached to poly-L-lysine coated coverslips and dehydrated in a graded acetone series. Samples were critical-point dried using CO_2_ as an intermedium. Specimens were coated with a carbon layer (SCD 500, Leica, Wetzler, Germany) and visualized with a MERLIN VP Compact field emission scanning electron microscope (Carl Zeiss, Jena, Germany) operated at the Electron Microscopy Center (EMZ).

### Cell wall protein extracts

Overnight culture bacteria were washed twice in PBS and resuspended in the protoplasting buffer containing 0.1 M KPO_4_ (pH 6.2), 40% sucrose, 10 mM MgCl_2_, and 10 units/ml of N-acetylmuramidase (Seikagaku Biobusiness, Tokyo, Japan). Incubation was performed for 3 h at 37°C. After centrifugation at 20 000 *g* for 10 min at 4°C, supernatants corresponding to the cell wall fractions were analyzed by SDS-PAGE and Western blot analysis.

#### Ethics approval statement

The protocol for the collection of human blood was approved by the Ethikkommission an der Medizinischen Fakultät der Universität Rostock (ethics committee vote: A 2014-0131). The experiments were conducted in accordance with the ICH-GCP guidelines. Informed consent was obtained from all subjects.
